# Living with Bats: The Case of Ve Golokuati Township in the Volta Region of Ghana

**DOI:** 10.1155/2017/5938934

**Published:** 2017-09-10

**Authors:** Jesse S. Ayivor, Fidelia Ohemeng, Elaine Tweneboah Lawson, Linda Waldman, Melissa Leach, Yaa Ntiamoa-Baidu

**Affiliations:** ^1^Institute for Environment and Sanitation Studies (IESS), College of Basic and Applied Sciences, University of Ghana, P.O. Box LG 209, Legon, Accra, Ghana; ^2^Department of Sociology, School of Social Sciences, College of Humanities, University of Ghana, P.O. Box LG 72, Legon, Accra, Ghana; ^3^Institute of Development Studies, University of Sussex, Brighton BN1 9RE, UK; ^4^Department of Animal Biology and Conservation Science, College of Basic and Applied Sciences, University of Ghana, Legon, Accra, Ghana; ^5^Centre for African Wetlands, University of Ghana, P.O. Box LG 67, Legon, Accra, Ghana

## Abstract

Transmission of zoonotic pathogens from bats to humans through direct and indirect contact with bats raises public apprehension about living close to bats. In the township of Ve Golokuati in Ghana, several “camps” of* Epomophorus gambianus* roost in fruit trees that provide ecosystems services for residents. This study explored human-bat interaction in the township and the potential risks of disease transmission from bats to humans. Data were derived through questionnaire administration and participatory appraisal approach involving focus group discussions, participatory landscape mapping, and transect walk. The study found that most human activities within the township, such as petty-trading, domestic chores, and children's outdoor recreation, exposed people to bats. Though there have been no reported cases of disease spillover from bats to humans from the perspective of residents and from medical records, respondents whose activities brought them closer to bats within the township were found to be more likely to experience fevers than those who do not interact with bats frequently. The study recommends education of community members about the potential risks involved in human-bat interactions and makes suggestions for reducing the frequent interactions with and exposure to bats by humans.

## 1. Introduction

In both urban and rural Ghana, two species of frugivorous bats, the Gambian epauletted fruit bat* (Epomophorus gambianus)* and the straw-coloured fruit bat* (Eidolon helvum)*, typically live close to humans [1, 2]. In the township of Ve Golokuati, the Afadzato South District capital in the Volta Region of Ghana, several “camps” of* E. gambianus* roost in mango and other fruit trees that provide shade and other ecosystems services for residents. Concerns have been raised about the closeness of bats to humans because of the ability of the former to spread zoonotic diseases [3–7]. Viruses isolated from bats include lyssaviruses, severe acute respiratory syndrome (SARS) and related coronaviruses (SARS-related CoV), filoviruses, henipaviruses, and other paramyxoviruses [2, 8, 9]. Living in close proximity to bats enables people to benefit from the ecosystem services that bats provide and may, simultaneously, put them at risk of disease spillover, which may be referred to as “ecosystems disservice.” Hunting and processing of bats for consumption potentially expose humans to zoonotic pathogens from bats through bites, scratches, and body fluids, as well as aerosolization of saliva, faeces, and/or urine [10, 11].

Past research has documented evidence of devastating spillover events of both lyssaviruses [12, 13] and filoviruses [14, 15] from bats to people in Africa. Filoviruses, such as Lake Victoria Marburgvirus which recorded high fatalities between 1998 and 2000 in DR Congo [16] and the Ebolavirus, have been most widespread in Africa [9]. Ebola outbreaks have been recurring since the first major case in DR Congo in 1995, which affected more than 260 humans and caused 186 deaths, was reported [14]. The most recent outbreak of Ebola in West Africa in 2014 infecting about 27,898 people and causing 11,296 reported deaths [17] raised public apprehension about human-bat interactions. This is because the outbreak has been traced back to a single incident of a young child playing in the vicinity of a hollow tree frequented by bats [18], now identified as a potential source of spillover of the EBV [11, 14, 19]. The ability of bats to spread zoonotic diseases and the fact they live close to humans heighten the potential threat of disease spillover.

Notwithstanding these negative perceptions, bats play vital roles in providing ecosystems services. They are well known for their roles in seed dispersal, pollination, maintaining soil fertility, and aiding in nutrient distribution [6, 20, 21]. Muscarella and Fleming [20] noted that bats facilitate seed dispersal in clear-cut strips as a result of how they defaecate and spit out seeds during flight. They also observed that many fruit bats use one or more feeding roosts each night and tend to deposit the vast majority of seeds ingested far away from fruiting plants. Through this habit, bats help to maintain species diversity by introducing seeds from outside disturbed areas. Bats in general have further been identified as bioindicators through their sensitivity to climate change, environmental degradation, and contamination by agrochemicals and other toxins [22, 23].

Considering the ecological importance of bats, vis-à-vis their role as reservoirs of zoonotic pathogens, a social management dilemma arises as to whether to encourage cohabitation of humans with bats or to eradicate bats living close to humans. According to Somphou et al. [24], the lack of information regarding the patterns of cohabitation with animals in urban areas hampers the design of effective measures of disease prevention. The residents of Ve Golokuati find themselves under this circumstance, which prompted this study.

The genesis of cohabitation of bats and humans in Ve Golokuati and the complexities of this relationship in the township have not been documented, neither has there been any disease surveillance in the area. This study explored the various ways people come into contact with* E. gambianus* in Ve Golokuati and the residents' perceptions of disease transmission risks from bats to humans.

In general, the effects of settlement expansion on bats' habitats are not well understood particularly on a landscape scale. Gehrt and Chelsvig [25] suggested that heterogeneous urban areas represent islands of habitat for bats within larger landscapes. While some earlier studies indicated that urbanization results in decreases in diversity and abundance of bats [26], others believe that the prevailing conditions and the patterns of development in the urban periphery encourage bats to concentrate in those areas [25, 27]. Urbanization can thus support bat-related activities and enhance ecosystem services for humans. Ultimately, as most emerging diseases exist within an intricately balanced host-agent continuum among wildlife, domestic animals and human populations [28] changes to this ecosystem can lead to changes in disease transmission potential.

The* Epomophorus gambianus* are generally found in woodland and savannah areas, mostly in the tropical regions of Africa and Asia [29]. According to Fenton [30], they roost in large hollow trees, thick foliage, and tree canopies along stream banks and below the thatch of open sheds. They roost low in trees during the day and are not disturbed by the presence of people [30]. Apart from the nuisance created from noise [1], not much has been documented on how their proximity affects humans and, until recently, this was not considered to be a problem. In addition, there have been no records of any disease spillover from bats to humans in Ghana or in the study area. The Ebola outbreak in parts of West Africa in 2014, as well as its link to bats as a potential source of contagion [31], has heightened people's concern [18] about bat-human interactions and their sense of risk. Thus better understandings of the cohabitation of humans and wild animals and the potential implications for human well-being are required urgently.

The “host-parasite continuum” framework [28], which suggests that wildlife and domestic animals and human populations coexist and that disease spillover occurs within a finely balanced host-agent continuum, provides the basis for exploring bat-human interactions in this paper. The framework highlights the ways in which underlying factors such as agricultural intensification, translocation, and human encroachment are responsible for emerging infectious diseases (EID).

## 2. Study Area

**Figure 1:**
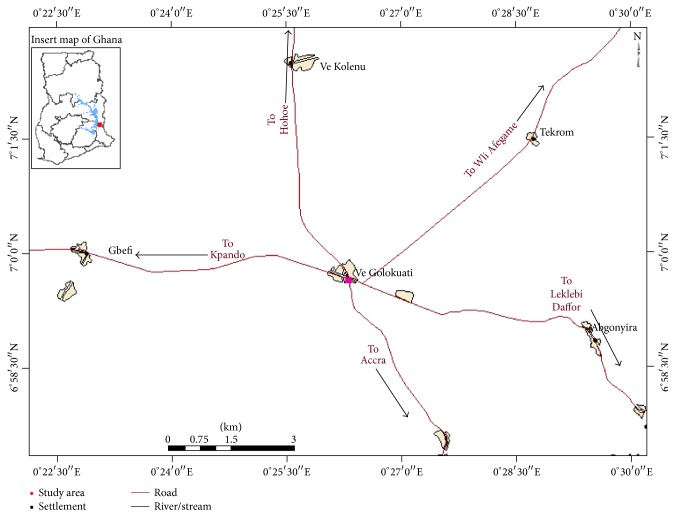
Map of the study area.

The study was carried out in Ve Golokuati, the district capital of Afadzato South District (Figure 1) between May 2013 and April 2015. The district lies in the wet semiequatorial climatic zone of Ghana, with annual rainfall values ranging between 1,016 mm and 1,210 mm. The bimodal rainfall regime which used to start in April, with two peaks in June and September, appears to be changing to a unimodal regime starting from late April and ending in October.

The area experiences an average of four to five months of dry season. Temperatures are high throughout the year, ranging from 26°C in the coolest months to about 32°C in the hottest months. Mean monthly temperature is about 29°C [32]. The town is located in the forest-savannah ecotone dominated by Guinea Savannah Woodlands. However, anthropogenic drivers, notably land conversion through rotational farming, lumbering, fuelwood extraction, and bush burning, have degraded the forest cover considerably. The town has a population of about 6,000 people, dominated by the Ewe-speaking ethnic group of the Volta Region. It is a “nucleated” settlement with houses built closely together without any clear layout. Apart from the major roads that pass through the town from east to west and north to south (see Figure 1), a few streets exist. Houses and compounds are separated by winding footpaths. Residents share facilities such as public places of convenience, water sources, and the open spaces such as recreational fields.

*Epomophorus gambianus *was the most dominant bat species in the study area, but, in a few cases, small numbers of other species such as the straw-coloured fruit bat* (Eidolon helvum)* and Peter's dwarf epauletted bat* (Micropteropus pusillus)* were observed. The* M. pusillus and E. gambianus *both occur in the township and are distinguished mainly by their facial looks and size, with* M. pusillus *being smaller. Also* M. pusillus* roost mostly in the lower parts of the same trees as* E. gambianus*. Residents believed that the two species were the same and that the separation was by age, but ecological studies carried out as part of our general studies on bats, which involved capturing of the bats, confirmed that indeed they were two different species. According to the residents, the bats migrated to Ve Golokuati in mid-to-late 1990s. It was, however, not clear where the bats came from. Whereas some believed that the bats migrated from Wli Water Fall area, a Wildlife Sanctuary located about 25 km from Ve Golokuati, others were of the view that the bats came from nearby bushes which were no longer habitable because of disturbances, notably incessant bush fires and agricultural conversion.

The congregation of bats in the township, according to the residents, has been enhanced by the presence of trees, particularly fruit trees, most of which have large crowns and provide shade as well as other ecosystems services to residents. The most common trees in this category, which also hosted colonies of bats, were mango* (Mangifera indica)* and neem* (Azadirachta indica)*. The fruits of these trees served as important sources of food for* E. gambianus,* although the bats are known to feed on fruits such as guava* (Psidium guajava)*, soursop fruit* (Annona muricata)*, whistling pine fruit* (Casuarina equisetifolia)*, cashew* (Anacardium occidentale)*, and pawpaw* (Carica papaya)* [33].

## 3. Methods

Both quantitative and qualitative data were used for the study. Quantitative data were derived from questionnaire administration among 150 residents of the study community through purposive sampling. Respondents targeted for interview included those in households located close to bats' roosts and individuals who through their daily activities such as farming, trading, recreation, and relaxation get exposed to bats either directly or indirectly. Most of these individuals were identified through snowball sampling where study subjects helped in the identification of other subjects. Respondents ranged in age between 18 and 75; 59% were females and 41% males. The questions posed to respondents centred mostly on activities that brought residents close to bats and whether or not respondents experience fevers and other strange diseases as a result of this exposure.

Participatory appraisal approach [34] was employed to solicit information on community perceptions of human-bat interactions and whether or not there were any health related issues as a result of such interactions. The approach involved participatory mapping, interviews, a transect walk, and focus group discussions (FGDs). A total of 13 elders and opinion leaders, ranging in age from 35 to 75 years, participated in the mapping exercise. The selection was done with the help of the Assemblyman for the area, who had a fair knowledge of individual competences. The exercise was informative and served also as a guide in determining the proximity of bats' roosts to residential areas and the selection of households for questionnaire administration. It also helped the research team to design a transect line to facilitate the transect walk that was undertaken following the mapping exercise.

**Figure 2:**
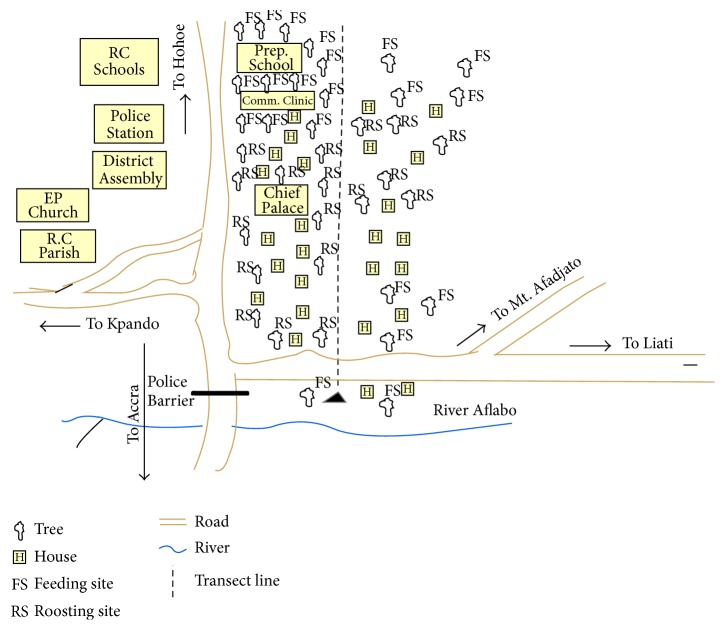
A sketch map of parts of Ve Golokuati showing the transect line.

A six-member team including one principal investigator undertook a transect walk from one end of town to the other (Figure 2). The walk was to identify the roosting and feeding sites of bats, the proximity of residential areas to these sites, and whether or not residents were exposed directly or indirectly to bats. During the walk, major landmarks ca. 50–75 m astride the transect line were noted. The exercise presented a further opportunity for the selection of households and other target groups for interview. A total of 18* E. gambianus* roosts and 19 feeding sites were observed along the transect line. Figure 2 shows the pattern of cohabitation of bats and humans in Ve Golokuati.

In addition, three focus group discussions were conducted separately for men, women, and opinion leaders of the town. Focus group discussions are a specific technique that uses the services of a facilitator to moderate a small group discussion. Each focus group discussion involved between eight (8) to 12 participants. A total of 35 people comprising 15 men and 20 women participated in the focus group discussions. The selection of participants followed a similar process as what was used in participatory mapping. Participants were between the ages of 25 and 76 and were made up of residents who had lived in the town for more than 15 years. The discussions were led by a facilitator and centred on the occurrence of bats in the town, local perceptions and attitudes towards bats, and perceived risk of disease spillover from bats to humans.

On-site observation took the form of visits to

- bats' roosting sites to ascertain the means by which human and bats interact;
- the outskirts of town to determine the occurrence of bats outside households where there was less protection;
- local market centre to investigate whether or not bat meat was sold;
- public places including the chief's compound and premises of basic schools where trees colonized by bats provided shade.

In all the places visited, informal interviews were held randomly with residents to understand human-bats interaction. On-site observation involved also the identification of trees in which bats roost and fruit trees that they feed on within the township. The researchers' own experience and local people's knowledge of native trees helped in the identification of the trees.

The study also solicited information from health facilities on types of common disease prevalent in the area. The research team had access to information on the top 20 cases that accounted for out-patient morbidity for the Afadjato North District and monthly out-patients morbidity returns for selected months from 2012 to 2014 from the District Health Directorate and the Ve Golokuati Health Centre, respectively.

There was a follow-up visit to the study area in April 2015, after the outbreak of Ebola virus in parts of West Africa. During this visit, 27 community members including the chief and elders of the town were engaged in a village square discussion [35] to find out about their perceptions and relationship with bats after the Ebola outbreak.

Data analysis used descriptive statistical tools such as statistical tables, graphs, and percentages to describe how the various variables relate. Multivariate logistic regression analysis was carried out to determine the association between experience of fevers and closeness of respondent's activity to bats.

## 4. Results

### 4.1. Demographic Information and Human-Bat Interaction

The findings showed that about 48% of the residents were migrants from over 40 different towns and villages in the Volta Region of Ghana. Among the respondents, 8% had tertiary education, 31% secondary education, 42% junior secondary/middle school education, and 19% little or no education. With regard to occupation, 75% of the respondents were farmers, while the rest were engaged in petty-trading (8%), fashion designing (3%), and other artisanal activities (2%). About 12%, mainly those above 65 years and those below 20, claimed that they were unemployed.

The focus group discussions backed by on-site observation through transect walk confirmed that human-bat interaction in the township was underpinned by the solid greenery that characterised the landscape by virtue of assortment of trees, which provided ecosystem services for humans and habitat and food for bats. Ecosystems services provided by the trees to residents in the township included food, medicine, shade, wind breaks, aesthetic beauty, and enhancement of microclimate. Moreover, the fact that the bats were hardly disturbed by residents made the township a protected niche for peaceful coexistence of bats and humans.

Though the exact number of the* Epomophorus gambianus* in Ve Golokuati is not known, it was estimated that over 5,000 of these bats lived with the people in the township. The occurrence of the bats, according to some participants at the focus group discussion, was the result of habitat disturbance, particularly wild bush fires and prolonged droughts in the mid-1980s. Additionally, the annual bush burning which precedes the farming season in the area, coupled with agricultural expansion that had taken over bat's habitats, had also contributed to the migration of the bats into the township. Respondents believed that the population of bats present in Ve Golokuati normally increased during the dry season (November to January), which coincided with the fruiting of plants, and decreased in the planting season (April to June). In spite of these seasonal fluctuations, there has been net decrease in total bat population in the town over the past decade from the perspective of respondents.

The results from the fieldwork showed that residents of the township came into contact with bats directly or indirectly in various ways. These included living in households with trees where bats roost, carrying out various vocations under trees, hunting of bats, and eating bat bushmeat. With regard to households, it was revealed that several of the residents carried out their daily chores outdoors under trees colonized by* E. gambianus* (Figures 5 and 6). Household chores that are noteworthy in relation to exposure to bats included food preparation, dish washing and manual laundry activities, and serving and eating food under trees. Participants in the focus group discussions revealed that when fruits on mango and guava trees ripe, household members may collect the partially eaten fruits discarded by bats as waste with their bare hands. It was further revealed that several households had installed rain harvesting facilities in the buildings to collect water for domestic uses such as washing and cleaning. The discussants indicated that the harvested rainwater was sometimes discoloured as a result of bat droppings, yet they found nothing wrong with the water.

Other daily activities that brought people into contact with bats were economic activities including farming and petty-trading at the market centre. On petty-trading, apart from a few traders who had their wares displayed in stalls, most of the traders plied their trade under mango trees, which incidentally also hosted large colonies of bats. Foodstuffs were sold in their raw state on the bare ground. All these activities invariably exposed both traders and buyers constantly to bats' urine and faecal matter, as well as to irritating bat behaviours such as dropping of foliage as they move from one branch to the other.

During focus group discussion, one petty trader who operated under one of the trees at the market reported the following:

The bats are a real nuisance to us. I was once eating under a mango tree at the market when I felt a substance dropping on my lower lip. It turned out to be a bat's dropping which compelled me to abandon my food even though I was hungry.

In some of the local school premises, school pupils, according to participants at FGD, engaged in outdoor recreational activities under trees that harbored several bat colonies. Another vantage location of exposure to bats and to bats' activities was the chief's palace, where mango trees providing shade for public gathering are colonized by* E. gambianus*.

### 4.2. The Use of Bats as Food

The use of bats as food brought people more directly into contact with bats than any other activity. The sequence of events involved in preparing bat meat as food, according to the findings, includes hunting, handling, butchering, dressing, smoking, and cooking. In the process, people come into contact with the blood of bats in addition to eating of the meat itself. With regard to the use of bats as food, a multivariate logistic regression analysis showed that there was 98% chance of more men eating bat bushmeat than women. This is statistically significant and may be attributed to some cultural prohibitions on bat consumption by pregnant women. For instance, the focus group participants believed that bats have no rectum and, therefore, vomit their excreta. For this reason, pregnant women were not allowed to eat bats; else they risked transmitting the “vomiting habit” to their babies after birth. Among the women respondents who claimed that they ate bat bushmeat, none of them had done so when they were pregnant because of this belief, yet this did not prevent women in general from eating bat bushmeat, as one middle aged woman disclosed:

I have always enjoyed bat meat because it is more delicious compared to other types of meat.

Participants at FGD indicated that no bat meat was offered for sale at the local market, though one could occasionally come across smoked* Eidolon helvum* bushmeat in other markets such as Kpando, about 15 km away.

Investigation into the ages of people who hunted and ate bat bushmeat revealed that it was mainly the adults that were involved (Table 1). The people of Ve Golokuati, however, would hardly eat* E. gambianus* but rather* E. helvum* which used to be abundant in the area in the past but has become rare locally. This would explain why most of the younger people did not eat bat bushmeat, since the* E. gambianus* which were abundant at the time of the study in the area are not appealing to most people because of their smaller size.

The results show further that only a small number of people across all the ages hunted for bats in the area. However, with regard to eating of bat bushmeat, people of age 45 and above, representing 16.7% of the total number of respondents, were involved. This was followed by those between 36 and 45 years representing 5.9%. However, none of the younger people (ages 15–25) interviewed ate bat bushmeat. An analysis of the relationship between eating of bat bushmeat and age was statistically insignificant (Chi square = 11.835, df = 12, and *P* = 0.459).

### 4.3. Other Forms of Human Exposure to Bats

Another way in which people came into contact indirectly with bats was through the aerial movements of bats between feeding and roosting sites. The bats reportedly moved on a daily basis between 5:30 pm and 6:00 pm and again between 4:30 am and 5:00 am. During these times when thousands of bats are in flight, the risk of contaminating water harvesting systems by bat through aerosolization of bat droppings is very high.

The focus group discussion further revealed that domestic stock (e.g., sheep, goats, and poultry) also interacted both directly and indirectly with the bats. Most of these animals were sheltered in pens and coops within the households but were allowed to roam in search of food during certain times of the day when they fed on foliage and fruits dropped by bats (also see [36]). Pets, notably cats and dogs, live within households but are also left to roam about freely within the township to hunt and scavenge for food. The threat of exposure to disease, through human-bat interactions, is reinforced by the predatory behaviour of the pied crow* (Corvus albus)* which targets the young* E. gambianus* just as they are being weaned. According the respondents, the crows often left bat carcasses which were often scavenged by dogs. This is noteworthy because, in the event of any disease spillover, transmission via domestic animals cannot be discounted.

**Figure 3:**
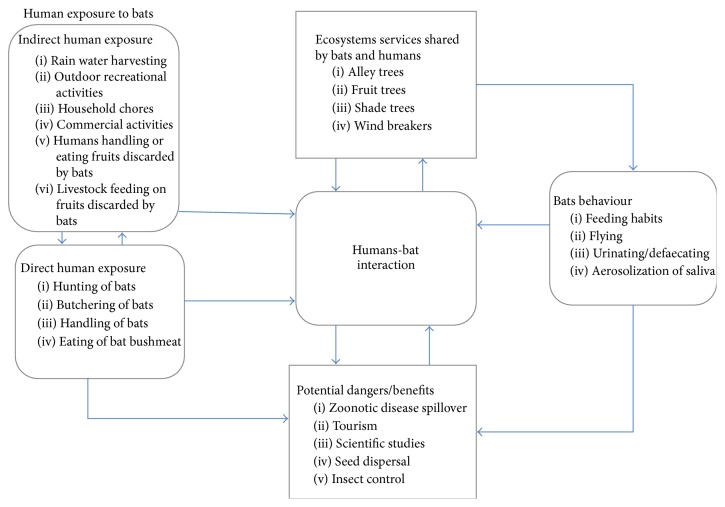
Schematic model showing human-bat interactions in the study area.

According to the findings, while most residents of Ve Golokuati appreciated the ecological role of bats and were willing to live with bats, others viewed them as awful animals whose activities, such as noise-making and contamination of harvested rain water through defaecation and littering, caused a lot of nuisance. Some of the residents only tolerated the presence of the bats because of the hope that the bats would bring some benefits such as ecotourism or research programmes to the community in the future and create employment opportunities in the township.  Figure 3 is a schematic model that demonstrates the human-bat interaction in Ve Golokuati. The figure illustrates the ecosystems services used by humans, activities of bats that bring them into contact with humans, human actions and activities that expose them to bats, and the potential dangers, as well as benefits of these interactions.

### 4.4. Possible Health Effects and Disease Spillover

Surprisingly, given their past experiences, the constant exposure to bats, and the media coverage of bats and Ebola, the residents of Ve Golokuati were strongly convinced that bats do not harbor any diseases and that there has not been any disease spillover from bats to humans. Their conviction came from the fact that there had not been any strange disease outbreaks in the town since the arrival of the bats.

Our investigations into disease prevalence and common causes of human deaths in the area during focus group discussions showed that malaria, locally known as* “Ndɔgbe,”* was the most common (note that there is a possibility of wrong diagnoses as this assessment is based only on local perceptions).

**Figure 4:**
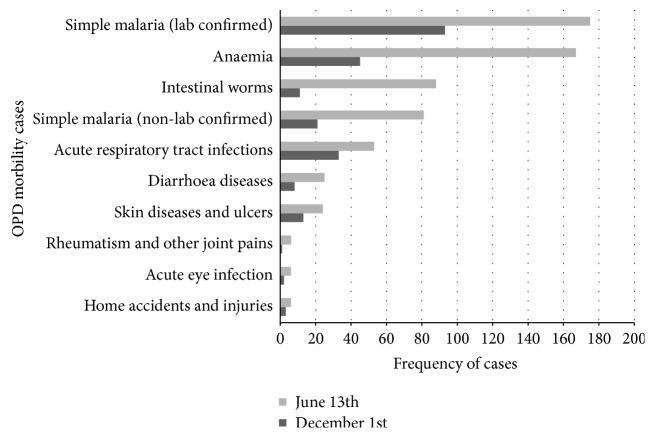
Top 10 OPD morbidity cases, Ve Golokuati Health Centre.

**Figure 5:**
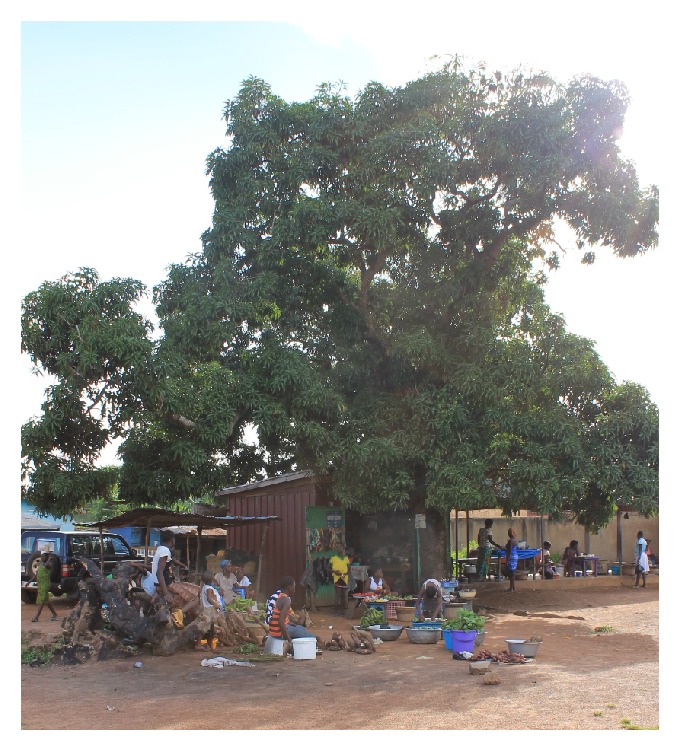
Petty traders operating under a mango tree used as roost by* E. gambianus*, Kofi Amposah©.

**Figure 6:**
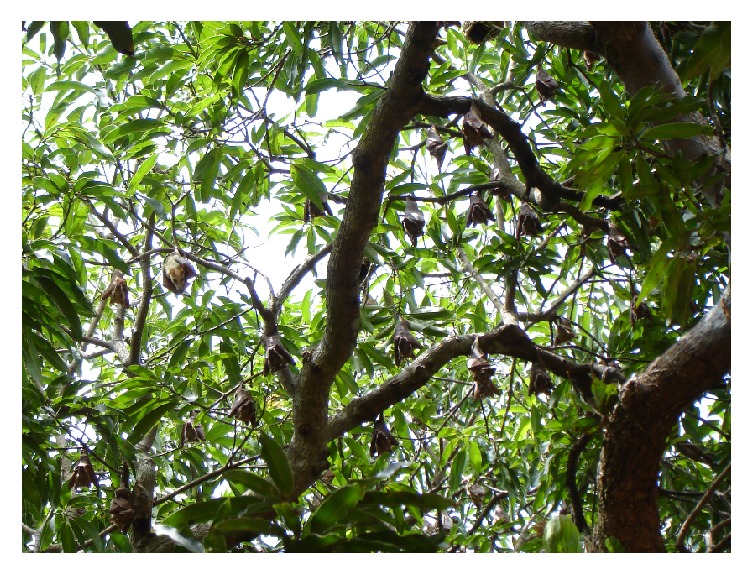
Scores of* E. gambianus *roosting in a mango tree over the market place J. S. Ayivor©.

The medical records obtained from the Out-Patient Department (OPD) of Ve Golokuati Health Centre corroborate the finding from the focus group discussions. The most common morbidity cases handled by the health centre were malaria, anaemia, intestinal worms, acute respiratory tract infections, diarrhoea, and skin diseases (Figure 4). The results indicated that, in all cases, the frequency of disease was higher in the rainy season (June) than in the dry season (December).

A further investigation into disease records from the District Health Directorate of the Afadzato South District showed that there were no records of any health condition specifically associated with bats in the district (Table 2). Among the top 10 diseases recorded by the District Directorate in 2013, malaria accounted for about 42% followed by acute respiratory infection (18%) and anaemia (9.7%). Though the medical records from health facilities in the area might not have recorded any zoonotic diseases, one cannot discount misdiagnosis since state-of-the-art equipment for diagnosing zoonoses may not be available in these facilities.

We assessed the experience of fevers among respondents in relation to the closeness of their economic activities to bats. The analysis showed that the various activities of the respondents were significantly associated with the experience of fevers (*P* < 0.05). From a multivariate logistic regression output (Table 3), respondents whose activities brought them close to bats were more likely to experience fevers than individuals whose activity were not close to bats.

Also farmers in the community who encountered activities of bats such as feeding on their farms were more likely to contracting fevers than those who do not encounter bats (odds ratio of 6.826 and coefficient of 1.921).

*E. gambianus *in the study area faced several threats, such as habitat destruction, predation, and occasional hunting by humans for bushmeat. The threats identified in this study confirm what the literature summarizes as human disturbance and habitat destruction [37, 38]. These threats were rooted in misinformation and incitement of people against the existence of bats. The outbreak of Ebola in parts of West Africa and the ensuing negative reportage about bats had led to the destruction of several trees to get rid of bats in the township.

## 5. Discussions

### 5.1. Human-Bat Interactions

The fundamental question underpinning the presence of* E. gambianus* in the township of Ve Golokuati is whether or not it is safe for people to live in close proximity to bats. The question as to how long humans have been interacting with bats in the township is also important as this may help in understanding the disease dynamics as portrayed by the medical records. Indeed, the genesis of occurrence of bats in the study area is not very certain, but research has shown that, generally, bats relocate in response to a range of environmental factors [39, 40]. According to Rebelo et al. [41], climate change has the potential to reduce the suitability of bat roosting sites, particularly tree roosts. This corroborates the claims by respondents that habitat disturbance caused by annual bush burning and agricultural conversion was the prime cause of relocation of bats into the township.

Cohabitation of humans and bats, as was the case in Ve Golokuati, was possible because the township contained trees that provided ecosystem services enjoyed by both humans and bats and because the bats provided vital ecosystem services to humans. Furthermore, the bats within the township were protected from threats and other disturbances better than in the wild. In a number of locations in Ghana where bats occur, they are under some form of protection for various reasons such as traditional taboos, military presence, or deliberate protection [42]. Human-bat interaction occurs for a number of reasons. Hayman et al. [7] observed that such interaction occurs because humans are encroaching on bats' habitats, just as bats are utilizing human structures as roosts. The interactions may be indirect, in the form of odour from faecal droppings, urine, aerosolization of saliva, and glandular body secretions [43]. It may also be direct through hunting and processing of bat bushmeat for consumption [2, 10]. In Ve Golokuati, the findings showed that human interaction with bats is both direct and indirect, which suggests that the residents may be at risk to disease spillover. The “host-parasite continuum” framework [28], suggests that wildlife, domestic animals, and human populations coexist and that disease spillover occurs within a finely balanced host-agent continuum. The framework highlights the ways in which underlying factors such as agricultural intensification, translocation, and human encroachment are responsible for emerging infectious diseases.

Though only a few people ate bat bushmeat in the study area, this form of direct interaction could easily spread disease pathogens among the people. Shakespeare [44] discounted risk of disease transmission through eating of bat bushmeat since the meat is normally well cooked but indicated that the risk of transmission may occur during capture and slaughtering of bats, during which time infection could occur through blood and body fluids or bites and scratches.

Currently there are no suspected cases of spillover of disease from bats to humans in Ve Golokuati. Though this might be good news to the residents, spillover of diseases cannot be completely ruled out since no systematic surveillance has been done in the Ve Golokuati Health Centre, which lacks the capacity in terms of personnel and equipment to do so. Furthermore, most suspected cases of fevers are treated as “malaria” and there is a possibility of misdiagnosis. In real terms, the official figures and respondents' perceptions may overdiagnose malaria, while overlooking and underestimating the endemic zoonotic disease burden [45, 46]. While attention is currently focused on bats and their potential to infect humans with Ebola and other zoonotic diseases such as henipaviruses, the limitations and difficulty to properly diagnose these diseases appear to be off the public health radar. This therefore calls for great caution as humans live with wildlife.

## 6. Conclusions

The presence of bats in the Ve Golokuati Township and their potential danger to humans in the face of emerging infectious disease pose a serious conservation dilemma. The findings suggest that residents of the township had been living with about 5000* E. gambianus* over the past two decades, and so far, there had been no suspected cases of disease spillover from bats to human. However, the documented potential epidemiological impacts of close human-bat interaction, particularly the recent outbreak of Ebola virus in parts of West African, give cause for concern. While getting rid of the bats by destroying the trees in Ve Golokuati may not be environmentally and ecological prudent, the question of how to reduce bat-human interaction to minimize the risk of any potential future disease spillover still remains.

A well-designed agenda for the relocation of the bats to surrounding forested areas with the help of experts from the Ghana Wildlife Division may offer a long term solution to curtail the regular interactions and exposure of humans to bats. Yet, relocating bats is not always easy, as demonstrated by the attempts at 37 Military Hospital in the capital city of Accra, where thousands of bats congregate (see [47]). Nonetheless, establishment of a forest grove close to the town, as a protected area, should be an option to facilitate a possible relocation. This would provide a protected space for the bat population and will, hopefully, decrease their frequent interaction with humans. The initiative would also help to promote ecotourism and meet the aspirations of the people.

In the short term, the contamination of harvested rainwater by bat urine and faeces can be minimized by delaying rainwater harvesting after long spells of absence of rains. By doing so, bats droppings on roof tops would be washed away before water harvesting starts. Residents also need to be educated on the need to avoid drinking untreated harvested rainwater. In order to reduce human exposures to bats, traditional leaders including the chief and elders may have to enforce prohibitions on hunting of bats to minimize disturbance and to create a safe haven for the bats. In the long term, improved diagnostics and systematic surveillance of zoonotic disease are required, coupled with regular health screening of the residents of Ve Golokuati, as well as other communities in Ghana where bats live in close proximity with humans.

Finally, the Health Directorate together with the District Assembly should sensitize the residents and, in the future, possibly tourists coming to see the bats, on the potential dangers of handling of bats, both dead and alive, and on appropriate ways to promote hygienic and good health practices such as regular hand washing, reporting all strange ailments promptly to medical centres and minimizing direct contact with bats through hunting, handling, and eating.

We recommend future research on water quality of both harvested rain water in the township and the nearby stream, which is the main source of potable water for the township.

## Figures and Tables

**Table 1:** Relationship between age and hunting/eating of bats meat.

Age range of respondents	Percentage of those engaged in bats hunting (*n* = 29)	Percentage of those who used bats as food (*n* = 29)
15–25	0	0
26–35	0.9%	4.6%
36–45	1.0%	5.9%
46–55	1.8%	14.4%
55 and above	0.8%	2.3%

**Table 2:** A year trend top 10 diseases seen at OPD 2013 (Afadzato South District).

Disease	Cases	%
Malaria	20761	41.8
Acute respiratory infection	9046	18.2
Anaemia	4819	9.7
Rheumatism & other joint pains	4283	8.6
Diarrhoea diseases	3304	6.7
Intestinal worms	3043	6.1
Skin diseases & ulcers	2420	4.9
Home accidents and injuries	799	1.6
Pneumonia	744	1.5
Typhoid fever	420	0.9

**Table 3:** Multivariate logistic regression analysis on experience of fevers associated with closeness of economic activity to bats.

Characteristics		Closeness of activity to bats	95% CI	*P* value	Odds ratio
Farming	Experience of fevers	1.921	(1.333, 34.950)	0.000^*∗*^	6.826
Trading	Experience of fevers	0.001	(0.968, 1.035)	0.000^*∗*^	1.001
Tailoring/dress making	Experience of fevers	0.001	(0.936, 1.071)	0.000^*∗*^	1.001
Artisanal	Experience of fevers	0.001	(0.936, 1.071)	0.000^*∗*^	1.001

^*∗*^Significant  *P* values (*P* < 0.05).
